# The Impact of Compulsivity and Impulsivity in Cerebellar Ataxia: A Case Series

**DOI:** 10.5334/tohm.550

**Published:** 2020-10-16

**Authors:** Nadia Amokrane, Chi-Ying R. Lin, Natasha A. Desai, Sheng-Han Kuo

**Affiliations:** 1 Department of Neurology, Columbia University Medical Center, New York, NY, US; 2 Initiative for Columbia Ataxia and Tremor, Columbia University Medical Center, New York, NY, US

**Keywords:** cerebellar ataxia, cerebellum, compulsivity, impulsivity, reward

## Abstract

**Background:**

The cerebellum has recently been identified to have a key role in reward processing, and individuals with ataxia have been found to be more impulsive and compulsive as part of cerebellum-related cognitive and behavioral disturbances.

**Case Report:**

We reported five individuals with cerebellar ataxia who demonstrate impulsive and compulsive behaviors, including hobbyism, gambling, and compulsive medication use, to illustrate that these symptoms can be highly disabling.

**Discussion:**

These five cases provide examples of behavioral symptoms in cerebellar ataxia. Further investigations of the pathomechanism of these symptoms will advance our understanding of the cerebellum in cognition and behavior.

## Background

The cerebellum, in coordination with the cerebrum, governs a variety of motor and non-motor functions of the brain. Individuals with cerebellar degeneration often have both motor impairment, manifesting as cerebellar ataxia, and non-motor dysfunction, presenting as a constellation of symptoms called cerebellar cognitive affective syndrome [1,2,3]. In cerebellar cognitive affective syndrome, depression and emotional liability are often described [1,2,4]. However, the complex nature of cognitive and behavioral disturbances in individuals with cerebellar degeneration is not entirely clear.

Recently, basic science studies in animal models have led us to a better understanding of the cerebellum in reward processing. Specifically, Purkinje cells, the principal neurons in the cerebellum encode the reward-related reinforcement learning in monkeys [5]. Purkinje cell activity is mainly regulated by two excitatory inputs: climbing fibers and parallel fibers. In mouse studies, climbing fiber activity is also closely linked to reward timing and prediction [6,7,8] whereas parallel fiber activity is related to the expectation of reward [9,10]. Optogenetic activation of cerebellar output sufficiently alters the reward behaviors in mice [11]. These studies provide mechanistic insight into how the cerebellum can modulate reward processing.

The next question is whether the cerebellum also plays a role in reward processing in humans. To test this hypothesis, we studied the classical symptoms related to abnormal reward: impulsivity and compulsivity, in individuals with cerebellar ataxia [12]. We found that individuals with cerebellar ataxia are overall more impulsive and compulsive than age-matched controls. In addition, they also exhibited domain-specific impulsivity and compulsivity in hobbyism-punding, gambling, and compulsive medication use [12]. The study provides supporting evidence that abnormal reward processing can occur in individuals with cerebellar ataxia.

However, we still do not have a clear understanding of whether and how these symptoms can impact the lives of individuals with cerebellar ataxia. Here, we reported five cases of cerebellar ataxia with prominent symptoms of impulsivity and compulsivity. Neither of these study subjects had a premorbid psychiatric disorder nor were they on psychiatric medications prior to the onset of the impulsive and compulsive behaviors.

## Case Reports

### Case 1

**Figure 1:**
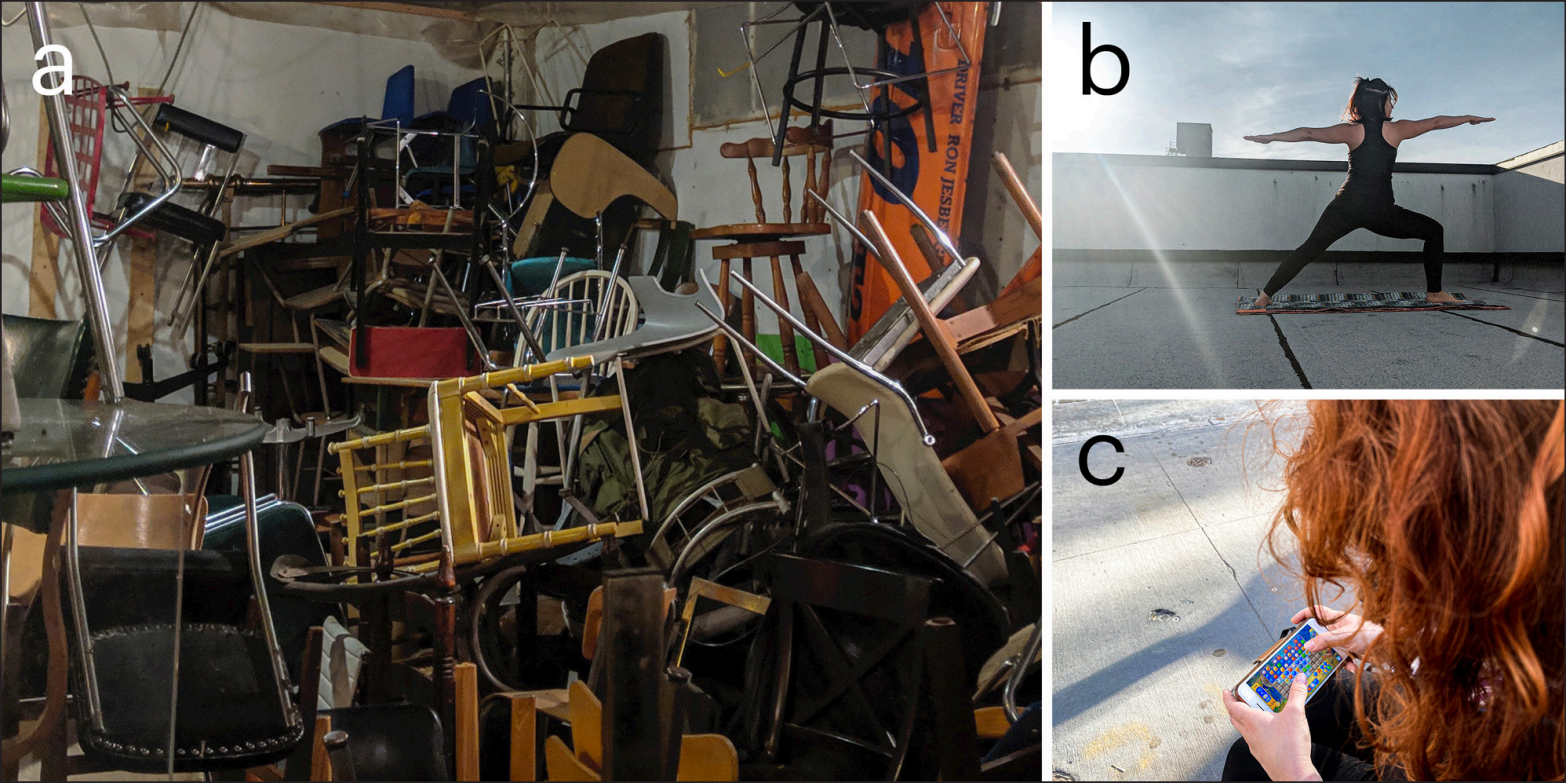
Impulsive and compulsive behaviors of cerebellar ataxia. **a:** hoarding for hobbyism-punding. **b:** excessive exercise. **c:** compulsive game playing.

A 49-year-old woman, a homemaker, has genetically-confirmed spinocerebellar ataxia type 2 (SCA 2). Her age of ataxia onset was at 27, and she now requires a walker to ambulate (Table 1). She developed compulsive thoughts at age 40, beginning with an obsessive idea that the end of the world is doomed to come after learning of the Rapture Doomsday 2011 event and Mayan calendar 2012 apocalypse. Since then, she started becoming a survivalist and prepper, and she has been hoarding excessive materials and supplies (Figure 1A), including water, canned foods, $2,000 worth of ammunition and guns and crossbows, two rain barrels for water collection, salt lick blocks (so that she could attract wild deer for hunting), and an electric generator with extra fuel stabilizer additive for long-term gasoline storage. She has dedicated the majority of the last decade to obsessively reading and researching on how to survive and be prepared for natural disaster events.

**Table 1:** Demographics, ataxia and impulsive compulsive symptom onset.

Case	Age	Gender	Diagnosis	Ataxia onset age	SARA	QUIP-RS score	Specific domain*	Duration of ataxia symptoms	Duration of impulsive and compulsive symptoms

Case 1	49	W	SCA 2	27	16	8	hobbyism/punding (hoarding/prepping)	22 years	9 years
Case 2	33	W	SCA 3	27	3	17	hobbyism/punding (exercise)	6 years	3 years
Case 3	38	W	SCA 1	27	15.5	45	hobbyism/punding (video games)	11 years	2 years
Case 4	53	M	SCA 2	49	14	8	gambling	4 years	3 years
Case 5	56	M	MSA-C	54	12	8	compulsive medication use	2 years	2 years

M = man; MSA-C = Multiple System Atrophy – Cerebellar type; QUIP-RS Score = Questionnaire for Impulsive-Compulsive Disorders in Parkinson’s Disease-Rating Scale; SARA = Scale for the Assessment and Rating of Ataxia; Specific domain* = Specific domain for impulsivity and compulsivity; SCA = Spinocerebellar Ataxia (genetically confirmed autosomal dominant ataxias); W = woman.

She is restless and anxious if supplies run low and frustrated and irritated if unable to replenish stock. The isolation and hoarding have contributed to an estranged relationship with her family. When family members comment on her hobby, she accepts that it could be inappropriate. She gets extremely uncomfortable if asked to stop or discontinue her hoarding and prepping behaviors.

In terms of impulsive behaviors, she has had episodes of binge eating since age 30 and she had severe weight gain, requiring a Lap-Band surgery. She also makes jokes with inappropriate sexual content that contribute to difficulties in social interactions.

### Case 2

A 33-year-old woman, a finance manager, has genetically-confirmed SCA3. Her age of ataxia onset was at 27 (Table 1). She still walks without assistance. Her compulsive thoughts and behaviors developed at 30 years old after her father passed away from SCA3. She has a strong fear of losing control of her body and mind. She compulsively engages in exercises with the hope to slow the progression of SCA3. She exercises daily, between 1–6 hours of vigorous workouts. She performs squatting, weightlifting, yoga, and high-intensity short-interval training. (Figure 1B) She spends around $800 a month on gym memberships, personal training sessions, and workout supplements. She bets against friends and family to competitive exercise challenges.

She also has impulsive traveling. Every few months, she has the urge to purchase a last-minute get-away flight. Her destinations are random and can be anywhere in the world. She disappears unexpectedly without notifying anyone during these travels, which results in significant concerns in her family as well as complicating her relationships.

### Case 3

A 38-year-old woman, a homemaker, with genetically-confirmed SCA1. Her ataxia symptoms started at 27 but she now walks with a walker. She developed compulsive symptoms at 36 (Table 1). She obsessively plays video games, specifically Candy Crush, on her smartphone (Figure 1C). She often plays 4–8 hours/day, 7 days a week. It is usually very difficult for her to stop playing when asked, which frustrates her husband because he needs help with chores. She gets annoyed when her behavior is commented on, and this has occasionally led to an argument. She has intrusive thoughts of playing video games on her phone when performing other activities such as making meals. She feels guilty about playing video games excessively instead of taking care of her family.

She also displays bouts of impulsive rage and aggression of verbal hostility as reactions to insignificant issues, such as when someone accidentally places her purse on her walker or when a sandwich bag was unexpectedly crumpled up. During these bouts, she has threatened divorce or moving away from the family.

### Case 4

A 53-year-old man, a retired cab driver, with genetically-confirmed SCA2. His ataxia onset was at 38. He now uses a cane to walk. He developed impulsive and compulsive symptoms at 40 (Table 1). His impulsive behaviors are betting on sports games and gambling in street craps. He enjoys the act of winning money. He admits occasionally lying and hiding the fact that he gambles from his partner or children. He is annoyed when his partner comments on his gambling. He regrets when he loses money and tries to recover it. He feels guilty and ashamed of his gambling habits, especially when he has lost money intended for other purposes. Even when he is not gambling, he often has thoughts of doing it and experiences difficulty stopping the thoughts. We thus conducted the Iowa gambling task on this subject. The number of his disadvantageous choices on deck A and B surpassed his advantageous choice on deck C and D close to 2.5-fold. This result further supports that he has risky behavior in decision making [13,14].

In addition, he frequently makes impulsive sexual jokes in inappropriate situations, such as during a doctor’s appointment and his jokes make healthcare staff feel uncomfortable. He also has extramarital affairs that resulted in the end of his marriage.

### Case 5

A 56-year-old man, a retired attorney, has multiple system atrophy cerebellar type (MSA-C). His ataxia onset is at age 54. His impulsive and compulsive symptoms started at age 54 as well. His compulsive thoughts center around medication use, particularly his dosing schedule.

He reports constantly thinking about the timing of the next dose of medication, and he worries about the location, timeliness, and dosing of his medication. While he takes his medications every 4 hours, he spends up to 5 hours a day organizing, discussing, and planning his medication use. Even after establishing a thorough treatment plan, he frequently adjusts the dose of his medications with an intent to achieve increased effectiveness. His compulsion leads him to simultaneously consult with 5 different Movement Disorders specialists across different states. His excessive questioning, consulting, and reassessment of his treatment plan creates tension with his wife as she gets frustrated with how much of her life is focusing on his care. He feels guilty about the burden that his compulsions put on his family.

Previously an avid gardener, he has had episodes of impulsively attempting to garden in his backyard despite his physical limitations and concerns from his family. He fell and moderately injured himself on two occasions prompting his family to not allow him in the garden and giving him a small indoor herb garden to substitute for a backyard.

## Discussion

The dopaminergic system, composed of pathways connected the prefrontal cortex, ventral striatum, and ventral tegmental area, traditionally considered to be responsible for the classical reward learning and processing [15]. Interestingly, from recent animal studies, the cerebellum was found to have direct or indirect connections with these brain regions [11]. In addition, the neuronal component within the cerebellum, including the granule neurons, climbing fibers, and Purkinje cell firing pattern, are associated with reward encoding and processing [5]. Along this line, we recently confirmed that behaviors associated with abnormal reward processing such as impulsivity and compulsivity are very common in patients with cerebellar ataxia [12].

In this case series, we have illustrated 5 examples of the compulsive and impulsive behavior in individuals with cerebellar ataxia. These examples support that the non-motor symptoms of cerebellar dysfunction can be highly disabling, even when ataxia severity is mild. These symptoms cause tension in inter-personal relationships loss of time and money, and are often neglected if not being questioned in the clinic.

One of the limitations of the current studies is the heterogenous causes for cerebellar ataxia. While we observed these compulsive and impulsive behaviors in SCA and MSA-C, these patients also have brainstem and/or basal ganglia involvement. Therefore, we cannot entirely exclude the possibility that these symptoms originate from the brain regions outside of the cerebellum. Future neuroimaging studies, such as the structural MRI of cerebellum and basal ganglia, are needed to clarify this limitation. Our prior study on quantitative assessment of compulsivity and impulsivity in cerebellar ataxias identified these behaviors in many ataxia patients, regardless of underlying causes [12]. Of note, our study also included patients with SCA6 and idiopathic late-onset cerebellar ataxia [12], which are considered to be of relatively “pure” cerebellar involvement.

We included the cerebellar cognitive affective syndrome scale, Beck depression index, and the Barrat impulsiveness scale of each case in Supplemental Table 1. In addition, we specifically conducted the Iowa gambling task on case 4, and the result shows that the choice of disadvantage responses outnumbered the advantage responses, consistent with decision-making problems [13,14,16,17].

Traditionally, impulsivity and compulsivity are implicated in the dysfunctional ventral tegmental area and basal ganglia, for which Parkinson’s disease is the prototypical disorder. Not only patients with Parkinson’s disease have neurodegenerative changes in these brain regions, additional levodopa use can also aggravate the dopamine dysfunction, collectively leading to abnormal reward processing [18] and associated impulsive and compulsive behaviors [19]. Recent neuroanatomical and physiological advances in animal models help us understand that ventral tegmental area could be directly modulated by the cerebellum [5,6,7,8,10,11]. Our study provides further evidence that the role of the cerebellum in reward processing in humans. Interestingly, Parkinson’s disease patients have impulsive and compulsive behaviors across all domains [20] whereas cerebellar ataxia patients exhibit domain-specific impulsive and compulsive behaviors [12], highlighting the differences in the mechanisms.

To our knowledge, there is no Food and Drug Administration approved pharmacological and non-pharmacological intervention to treat impulsivity and compulsivity.

Unfortunately, to our knowledge, there is no Food and Drug Administration-approved pharmacological and non-pharmacological interventions to treat impulsivity and compulsivity. However, fluoxetine, mood stabilizer/anticonvulsant, cognitive behavioral therapy, and psychodynamically informed psychotherapy might be effective and could serve as the potential empirical or therapeutic interventions. None of these cases received therapies specifically for impulsivity and compulsivity, partly because of the under-recognition of these disabling symptoms in individuals with cerebellar ataxia. Our case report thus underscores how these behavioral symptoms can have impacts on the quality of life for patients and their families, which will be the first step towards the improvement of care.

In conclusion, our present study demonstrates severe cases of compulsivity and impulsivity in cerebellar ataxia. Traditionally, cerebellar cognitive affective syndrome includes executive dysfunction, visuospatial dysfunction, verbal memory and language disturbance, and poor abstract reasoning. As to the behavioral issues, apathy, lack of social skills, poor emotional control, and disinhibition were identified [1,2,4]. Our previously published article [12] and present case series further support that compulsivity and impulsivity could be regarded as a broader cerebellar cognitive affective syndrome.

## Additional Files

The additional files for this article can be found as follows:

10.5334/tohm.550.s1 Supplemental Table 1. Cerebellar cognitive affective syndrome scale (CCAS-Scale), Beck depression index, and Barratt impulsiveness scale of the 5 individuals of the present case series.

10.5334/tohm.550.s2 Supplemental Table 2. The current medications of the 5 subjects with cerebellar ataxia.
