# Supplementary material for: The Impact of Compulsivity and Impulsivity in Cerebellar Ataxia: A Case Series
Source: Tremor Other Hyperkinet Mov (N Y). 2020 Oct 16;10:43. doi: 10.5334/tohm.550 (PMC7583703; doi:10.5334/tohm.550)
Supplement: Supplemental Table 1. — Cerebellar cognitive affective syndrome scale (CCAS-Scale), Beck depression index, and Barratt impulsiveness scale of the 5 individuals of the present case series. [file tohm-10-1-550-s1.pdf]

[1] M = man; MSA-C = Multiple System Atrophy – Cerebellar type; QUIP-RS Score = Questionnaire for Impulsive-Compulsive Disorders in Parkinson's Disease-Rating Scale; SARA = Scale for the Assessment and Rating of Ataxia; Specific domain* = Specific domain for impulsivity and compulsivity; SCA = Spinocerebellar Ataxia (genetically confirmed autosomal dominant ataxias); W = woman

Case	Diagnosis	Duration of illness since symptom onset (years)	CCAS*	Beck depression inventory II	Barratt impulsiveness scale
Case 1	SCA 2	22 years	107/120 & 0/10	17/63	4/120
Case 2	SCA 3	6 years	94/120 & 0/10	3/63	72/120
Case 3	SCA 1	11 years	85/120 & 2/10	5/63	71/120
Case 4	SCA 2	4 years	64/120 & 6/10	7/63	47/120
Case 5	MSA-C	2 years	74/120 & 4/10	20/63	61/120

*CCAS: cerebellar cognitive affective syndrome. The total score of the scale comprises two, one is the total raw score (total = 120) and the other is the total pass score (pass of each domain. = 1, failure of each domain = 0, total = 10); MSA-C = Multiple System Atrophy – Cerebellar type; SCA = Spinocerebellar Ataxia (genetically confirmed autosomal dominant ataxias)

Supplemental Table 1.

Cerebellar cognitive affective syndrome scale (CCAS-Scale), Beck depression index, and Barratt impulsiveness scale of the 5 individuals of the present case series.
