# Supplementary material for: The Impact of Compulsivity and Impulsivity in Cerebellar Ataxia: A Case Series
Source: Tremor Other Hyperkinet Mov (N Y). 2020 Oct 16;10:43. doi: 10.5334/tohm.550 (PMC7583703; doi:10.5334/tohm.550)
Supplement: Supplemental Table 2. — The current medications of the 5 subjects with cerebellar ataxia. [file tohm-10-1-550-s2.pdf]

| Case | Medication | Dose | Frequency |
|---|---|---|---|
| Case 1 | Alprazolam | 25 mg | as needed |
| | Vitamin B12 | 1,000 mcg | daily |
| | Ferrous Sulfate | 65 mg | daily |
| Case 2 | Norethindrone acetate/ethinyl estradiol | 1 mg /10 mcg | daily |
| | Ferrous fumarate | 10 mcg | |
| Case 3 | Troriluzole | 140 mg | daily |
| | Calcium | 1,200 mg | daily |
| Case 4 | CoQ10 | 600 mg | twice a day |
| | Brimonidine | 0.06 ng/mL | twice a day |
| | Vitamin C | 1,000 mg | daily |
| | Vitamin E | 15 mg | daily |
| Case 5 | Carbidopa/levodopa | 75mg- 100mg | Every 2 hours |
| | Baclofen | 15 mg | daily |
| | Gabapentin | 100 mg | daily |
| | Duloxetine | 20 mg | daily |
| | Methadone | 50 mg | as needed |
| | Tylenol | 325 mg | as needed |
| | Super B complex | 1 tablet | daily |
| | Vitamin D3 | 1,500 U | daily |

**Supplemental Table 2.**
The current medications of the 5 subjects with cerebellar ataxia
